# Different Tidal Volumes May Jeopardize Pulmonary Redox and Inflammatory Status in Healthy Rats Undergoing Mechanical Ventilation

**DOI:** 10.1155/2021/5196896

**Published:** 2021-10-29

**Authors:** Leandro da Silva Cândido, Natália Alves de Matos, Thalles de Freitas Castro, Laisy Cristina de Paula, Aline Maria dos Santos, Guilherme de Paula Costa, André Talvani, Silvia Dantas Cangussú, Walter Araujo Zin, Frank Silva Bezerra

**Affiliations:** ^1^Laboratory of Experimental Pathophysiology, Department of Biological Sciences, Institute of Exact and Biological Sciences, Federal University of Ouro Preto (UFOP), Ouro Preto, Brazil; ^2^Laboratory of Cardiovascular Physiology, Department of Biological Sciences, Institute of Exact and Biological Sciences, Federal University of Ouro Preto (UFOP), Ouro Preto, Brazil; ^3^Laboratory of Immunobiology of Inflammation, Department of Biological Sciences, Institute of Exact and Biological Sciences, Federal University of Ouro Preto (UFOP), Ouro Preto, Brazil; ^4^Laboratory of Respiration Physiology, Carlos Chagas Filho Institute of Biophysics, Federal University of Rio de Janeiro, Rio de Janeiro, Brazil

## Abstract

Mechanical ventilation (MV) is essential for the treatment of critical patients since it may provide a desired gas exchange. However, MV itself can trigger ventilator-associated lung injury in patients. We hypothesized that the mechanisms of lung injury through redox imbalance might also be associated with pulmonary inflammatory status, which has not been so far described. We tested it by delivering different tidal volumes to normal lungs undergoing MV. Healthy Wistar rats were divided into spontaneously breathing animals (control group, CG), and rats were submitted to MV (controlled ventilation mode) with tidal volumes of 4 mL/kg (MVG4), 8 mL/kg (MVG8), or 12 mL/kg (MVG12), zero end-expiratory pressure (ZEEP), and normoxia (FiO_2_ = 21%) for 1 hour. After ventilation and euthanasia, arterial blood, bronchoalveolar lavage fluid (BALF), and lungs were collected for subsequent analysis. MVG12 presented lower PaCO_2_ and bicarbonate content in the arterial blood than CG, MVG4, and MVG8. Neutrophil influx in BALF and MPO activity in lung tissue homogenate were significantly higher in MVG12 than in CG. The levels of CCL5, TNF-*α*, IL-1, and IL-6 in lung tissue homogenate were higher in MVG12 than in CG and MVG4. In the lung parenchyma, the lipid peroxidation was more important in MVG12 than in CG, MVG4, and MVG8, while there was more protein oxidation in MVG12 than in CG and MVG4. The stereological analysis confirmed the histological pulmonary changes in MVG12. The association of controlled mode ventilation and high tidal volume, without PEEP and normoxia, impaired pulmonary histoarchitecture and triggered redox imbalance and lung inflammation in healthy adult rats.

## 1. Introduction

Mechanical ventilation (MV) is an important therapeutic tool to restore or improve gas exchange in patients with respiratory failure [1–3]. Many subjects in intensive care units and operating rooms undergo MV every year. Although most subjects recover quickly, resulting in weaning from the ventilator, it is estimated that between 4 and 13% of them require mechanical ventilation for long periods [4–7]. MV can trigger ventilator-induced lung injury (VILI), an iatrogenic harm to patients without previous pulmonary involvement that may contribute to mortality [8, 9].

Volutrauma potentially triggers VILI, because of excessively high tidal volumes, thus leading to lung injuries [10]. Alveolar overstretching generated by high tidal volumes can cause alveolar rupture, lung damage, increased vascular permeability, release of inflammatory mediators, and, thus, inflammatory cell recruitment into the alveolar space [10, 11]. Neutrophils are the main leukocytes involved in acute lung injury and VILI [12], but it is not known whether once present and activated in the alveolar space, these cells can contribute to the production of inflammatory markers such as cytokines and reactive oxygen species [13].

To maintain cellular redox homeostasis, the body counts on specific biological defense systems, including antioxidant enzymes such as superoxide dismutase, catalase, and the glutathione system [14]. The imbalance between reactive species and antioxidant agents present in the lung tissue can cause oxidative stress [8, 15], damaging cellular metabolism regulation and oxidizing macromolecules such as DNA, proteins, and lipids [15, 16].

Since MV may damage healthy lungs, it is recommended to customize ventilator settings and constantly adjust them to match each individual's needs. Since MV itself causes severe comorbidities, the optimization of ventilation strategies is of paramount importance for the effective therapy of critical care patients, and, furthermore, proper MV requires a sound understanding of respiratory physiology, ventilator operational characteristics, and controlling complications associated with MV [17, 18]. Even though MV is frequently used, many intensive care units do not possess devices to adequately monitor it.

MV can cause lung injury [19, 20]. Hence, it is extremely important to immediately detect putative initial pulmonary changes to obtain the maximum benefit from the ventilatory strategy. To our knowledge, there is no information available on the putative association of redox imbalance and inflammatory status to convey lung injury. To explore this possibility, we assessed the outcomes of MV with different tidal volumes on otherwise healthy lungs.

## 2. Materials and Methods

### 2.1. Ethics Committee and Animals

Thirty-two 10-12-week-old male Wistar rats were obtained from the Animal Science Center of the Federal University of Ouro Preto (UFOP). The animals were kept in cages under controlled conditions of light, temperature, and humidity (12 h light/dark cycle, 24°C, and 50 ± 10%, respectively) and were provided water and food *ad libitum*. The animals received care according to the following guidelines: ARRIVE, National Council for Animal Experimentation Control, Ministry of Science, Technology, and Innovation (CONCEA/MCTI), Brazil, the “Principles of Laboratory Animal Care” formulated by the “National Society for Medical Research”, and the “Guiding Principles in the Care and Use of Animals” approved by the Board of the American Physiological Society. The Animal Ethics Committee on the Use of Animals (CEUA), Federal University of Ouro Preto, approved the present study (code: 2017/42).

The rats were randomly divided into four groups, 8 animals/group: spontaneously breathing control group (CG) and mechanical ventilation groups under three tidal volumes, namely, 4 mL/kg (MVG4), 8 mL/kg (MVG8), and 12 mL/kg (MVG12).

### 2.2. Spontaneous Ventilation

The control group spontaneously breathed room air. Subsequently, each animal was placed individually in a hermetically sealed body plethysmograph for 8 minutes to record respiratory rate, tidal volume, and minute ventilation. Medical grade compressed air (White Martins, São Paulo, Brazil) was flushed through the bodybox. Tidal volume was measured by the table spirometer (ADInstruments, Dunedin, New Zealand). The signal was amplified and analyzed using the PowerLab software (ADInstruments, Dunedin, New Zealand) [21].

### 2.3. Mechanical Ventilation

MVG4, MVG8, and MVG12 animals were intraperitoneally anesthetized with midazolam chloride (5 mg/kg) and fentanyl (0.16 mg/kg). Subsequently, neuromuscular blockade was produced by intravenous injection of suxamethonium chloride (1 mL/kg). The animals were placed on a surgical table in the supine position. The anterior cervical region was surgically opened, the musculature dissected, and the trachea exposed. The animals were tracheostomized, and a stainless-steel cannula (16 G) (Harvard Apparatus, Holliston, MA, USA) was indwelled into the trachea. The cannula was connected to a mechanical ventilator (Harvard Inspira Advanced Safety Ventilator, Harvard Apparatus, Holliston, MA, USA) under volume-controlled mode and tidal volumes (VT) of 4, 8, or 12 mL/kg, respiratory rate (RR) of 70 breaths/min, inspired fraction of oxygen (FiO_2_) of 21%, ZEEP, and inspiration/expiration ratio: 1 : 2. MV lasted 60 min [22].

At the end of MV, a 200 *μ*L blood sample was collected directly from the right femoral artery using a heparinized syringe (Monovette®, Sarstedt, Germany) for arterial blood gas analysis by the PRIME + ® VET gasometer (Nova Biomedical Corporation, Waltham, MA, USA). The animals were euthanized by an overdose of ketamine (130 mg/kg) and xylazine (0.3 mg/kg) [21].

### 2.4. Bronchoalveolar Lavage and Lung Collection

Immediately after euthanasia, the thorax was surgically opened, the left main bronchus clamped, the trachea cannulated, and the right lung was washed out with warm (37°C) saline solution (NaCl 0.9%). The procedure was performed three times, totaling a final BALF volume of 2.5 to 3.0 mL. The samples were stored in polypropylene tubes and kept on ice (4°C) to avoid cell lysis. BALF was centrifuged for 10 min at 4°C and 3,582 g (Eppendorf 5415R, Eppendorf®, Hamburg, Germany) for the evaluation of leukocytes in the air spaces. The supernatant was stored at -80°C, and the pellet was resuspended in 0.1 mL of saline solution. Twenty *μ*L of the resuspended solution was placed in a tube containing 180 *μ*L of Turk solution, which was used for total leukocyte count in a Neubauer chamber. Differential cell counts were performed on cytospin preparations (INBRAS Equipamentos para a Saúde, Jardinópolis, SP, Brazil) stained with a fast panoptic coloration kit (Laborclin, Pinhais, PR, Brazil) using standard morphological criteria to identify cell types. Briefly, 100 cells were counted per slide under an optical microscope at 100x magnification. Two domain researchers double-blindly counted unidentified slides at different occasions [21].

After BALF collection, the right ventricle was perfused with warm saline solution (NaCl 0.9%) to wash blood out of the lung circulation. Then, the right bronchus was clamped. The left lung was instilled with 4% buffered formalin (pH 7.2) under a pressure of 25 cmH_2_O for 2 min, securely closed, removed, and immersed in a fixative solution for 48 hours. The samples were routinely processed, and the slides were stained with hematoxylin and eosin (H&E) for stereological analysis (see below). The right lung was collected, and 100 mg of tissue was homogenized with 1 mL phosphate buffer (pH 7.8); the samples were centrifuged for 10 minutes at 4°C and 15,521 g; the supernatant was collected and stored at −80°C [21].

### 2.5. Immunoassays for Inflammatory Markers

Inflammatory markers, namely, interleukin-1 (IL-1), interleukin-6 (IL-6), chemokine (CC motif) ligand 5 (CCL5), and tumor necrosis factor alpha (TNF-*α*), were assayed in lung homogenate by Enzyme-Linked Immunosorbent Assay (ELISA) using commercial kits (PeproTech, Ribeirão Preto, Brazil) according to the manufacturer's recommendations. Immunoassays were performed in 96-well plates on which 100 *μ*L of monoclonal antibody to the protein (or peptide) of interest was added and samples were diluted in PBS containing 0.1% bovine serum albumin (BSA, Sigma-Aldrich, St. Louis, MO, USA). After incubation for 12 h at 37°C, the plates were blocked with 300 *μ*L/well of a PBS solution containing 1% BSA for 1 h at 37°C. Fifty *μ*L samples were added into each well. Reading used a wavelength of 450 nm [23].

### 2.6. Redox Analyses

Antioxidant enzymatic activity and oxidative damage were determined in lung tissue homogenates. Superoxide dismutase (SOD) activity was evaluated according to Marklund and Marklund [24], as the SOD ability to inhibit pyrogallol autoxidation. Catalase activity (CAT) was measured by the decrease in hydrogen peroxide (H_2_O_2_) according to Aebi [25]. Glutathione dosage was determined by an assay adapted from the commercial Sigma kit (# CS0260, Sigma-Aldrich, St. Louis, MO, USA), which uses a kinetic method to measure total glutathione (GSH + GSSG) by reducing 5.5′-dithiobis(2-nitrobenzoic acid) to thio-2-nitrobenzoic acid [26]. Lipid peroxidation was analyzed according to Buege and Aust [27]: thiobarbituric acid reacts with oxidized lipids and generates malondialdehyde. Carbonylated protein was analyzed according to a protocol adapted from Reznick and Packer [28]. Myeloperoxidase (MPO) content was measured using 3,3′,5,5′-tetramethylbenzidine (TMB), hexadecyl trimethyl ammonium bromide (HTAB), H_2_O_2_, and sodium acetate buffer (NaOAc) according to Campos et al. [29]. The enzyme activity was expressed as U/mg of total protein, whose content was determined according to Bradford [30].

### 2.7. Stereological Analyses

In order to assess pulmonary histoarchitecture, 20 randomly picked fields in histological sections were captured using a light microscope (Primo Star, Carl Zeiss, Oberkochen, Germany) equipped with the Axiocam 105 digital camera (Carl Zeiss, Oberkochen, Germany) driven by the ZEN lite image capture software (400x magnification) [23]. The volume density analyses of the alveolar septum (Vv [sa]) and alveolar airspace (Vv [a]) were performed in a test system composed of 16 points and a known test area, to avoid overestimating the number of structures. The test system was coupled to a monitor, and 20 fields were analyzed to obtain uniform and proportional lung samples. The number of points (Pp) that fell on alveolar septa (Vv [sa]) and spaces (Vv [a]) was counted according to the total number of points in the test system (Pt) as described by Mandarim-de-Lacerda [31] and Valença et al. [32].

### 2.8. Statistical Analysis

The normal distribution of each variable was examined using the Kolmogorov-Smirnov test; the homogeneity of the variances was evaluated by Bartlett's test. For comparison among groups, a one-way ANOVA followed by Tukey's post hoc test was used. Parametric data are presented as mean and standard deviation. For nonparametric data, we used the Kruskal-Wallis test followed by Dunn's posttest, and the data are expressed as median, 25 and 75% percentiles. In both cases, the difference was considered significant when *p* < 0.05. The statistical analyses were performed using Prism v.5 software (GraphPad Software, San Diego, CA, USA).

## 3. Results

### 3.1. Arterial Blood Gases

MVG12 animals presented pH, pCO_2_, and HCO_3_^−^ (mEq/L) significantly different from CG and MVG4 rats (Table 1): pH was higher in MVG12 than in CG, MVG4, and MVG8 rats (ANOVA; *p* < 0.0001); lower PCO_2_ and HCO_3_^−^ values in MVG12 than in the remaining groups (ANOVA; *p* < 0.0001). PO_2_, SO_2_, and PO_2_/FIO_2_ were lower in MVG4 than in CG, MVG8, and MVG12 rats (ANOVA; *p* < 0.0003, *p* < 0.0001, and *p* < 0.0008, respectively).

### 3.2. Pulmonary Function

In MVG4, MVG8, and MVG12 groups, RR remained unaltered resulting in proportional increases in minute ventilation. Therefore, MVG12 rats presented higher minute ventilation than CG, MVG4, and MVG8 groups (ANOVA, *p* < 0.0001, Table 1).

### 3.3. Influx of Inflammatory Cells into the BALF

**Figure 1:**
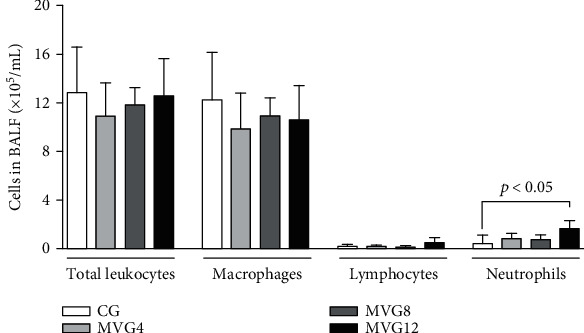
Inflammatory cells in bronchoalveolar lavage fluid (BALF). CG: control group; MVG4, MVG8, and MVG12: mechanical ventilation groups ventilated with 4 mL/kg, 8 mL/kg, and 12 mL/kg, respectively. Data are expressed as mean + standard deviation. ANOVA followed by Tukey's posttest (*p* < 0.05). *N* = 8 animals/group. Significantly different groups are indicated by horizontal square brackets and *p* values.

Total and differential inflammatory cell count in BALF after 1 h ventilation was done to verify their putative influx into the airways. A higher neutrophil count was observed in MVG12 rats than in CG (ANOVA; *p* < 0.005), as depicted in Figure 1.

### 3.4. Inflammatory Markers in Lung Tissue

Inflammatory cytokine and chemokine data are listed in Table 2. IL-1 was higher in MVG12 than in CG and MVG4 rats (ANOVA; *p* < 0.002). IL-6 was greater in MVG12 than in CG and MVG4 rats (ANOVA; *p* < 0.001). Similarly, TNF was larger in MVG12 than in CG and MVG4 rats (ANOVA; *p* < 0.007). CCL5 was smaller in MVG12 than in CG (ANOVA; *p* < 0.004).

### 3.5. Biomarkers of Oxidative Stress in Lung Parenchyma

Pulmonary homogenate revealed that MV with 12 mL/kg tidal volume damaged the lung (Table 3). Lipid peroxidation level was higher in the MVG12 group (ANOVA; *p* < 0.0009) than in CG, MVG4, and MVG8 rats. Protein carbonylation was also greater in MVG12 (ANOVA; *p* < 0.01) than in CG and MVG4 rats. The activity of superoxide dismutase was larger in MVG12 (ANOVA; *p* < 0.01) than in CG and MVG4, and catalase activity was higher in MVG12 (ANOVA; *p* < 0.03) than in CG rats. In addition, MPO activity was greater in MVG12 (ANOVA; *p* < 0.007) than in the CG group.

### 3.6. Stereological Analysis of the Lung Parenchyma

**Figure 2:**
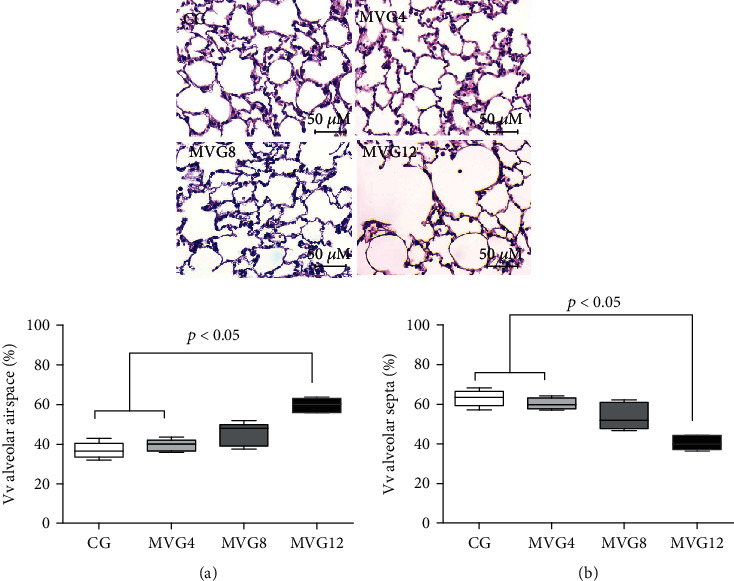
Histological images of lungs pertaining to all experimental groups (upper four panels). Stereological analyses of lung sections (lower two plots). CG: control group; MVG4, MVG8, and MVG12: mechanical ventilation groups ventilated with 4 mL/kg, 8 mL/kg, and 12 mL/kg, respectively. Volume density (%Vv) of the alveolar airspace (a) and alveolar septa (b). Photomicrographs of lung sections stained with hematoxylin and eosin. Bar = 50 *μ*m, 400x magnification. Data are expressed as median, 25% and 75% percentiles; whiskers encompass upper and lower limits and were analyzed by Kruskal-Wallis ANOVA followed by Dunn's posttest. *N* = 5 animals/group. Significantly different groups are indicated by horizontal square brackets and *p* values.

Our results demonstrate that MV with 12 mL/kg tidal volume for one hour caused alterations in the pulmonary architecture (Figure 2, MVG12), as evidenced by an increase in alveolar volume density (Vv [a]) (Figure 2(a)) and a decrease in alveolar septa volume density (Vv [sa]) (Figure 2(b)) when compared to CG and MVG4 (Kruskal-Wallis ANOVA; *p* = 0.0039).

Alveolar structure differed among groups (Figure 2). Vv [a] was greater in MVG12 (Kruskal-Wallis ANOVA; *p* = 0.0039) (60.12 (55.95–63.10)) than in CG (36.56 ((33.44–40.63)) and MVG4 (40.18 (36.41–42.11)) groups. As a result, the MVG12 Vv [sa] (39.88 (36.91-44.05)) was smaller than in CG (63.44 (59.22-66.57)) and MVG4 (59.82 (57.89-63.10)) rats (Kruskal-Wallis; *p* = 0.0039).

## 4. Discussion

Mechanical ventilation is important to save lives needing respiratory support. However, it is mandatory to constantly check the settings of the mechanical ventilator, because, if inadequately managed, it may trigger lung damage, which can overshadow the initial benefit and even lead to death [17]. Recently, lung-protective ventilation has been associated with low tidal volumes in the range of 6–10 mL/kg predicted body weight [33]. In this context, we used previously healthy animals and ventilated them with a tidal volume frequently used in clinical practice, i.e., a physiologic volume (8 mL/kg). Additionally, lower and higher tidal volumes (4 and 12 mL/kg, respectively) were tested in other groups of rats. A control group of spontaneously breathing rats was also included in the study. We found that mechanical ventilation with the highest tidal volume recruited inflammatory cells into the lungs, increased inflammatory markers and oxidative stress, and damaged the lung parenchyma.

Mechanical ventilation with high tidal volumes, approximately from 15 to 45 mL/kg, stretches lung tissue and impairs pulmonary function [34]. However, we disclosed significant pulmonary alterations using volumes close to those considered as noninjurious (6-10 mL/kg) [33]. The comparison of gas exchange among the ventilated groups revealed that the group ventilated with the highest tidal volume, although importantly impairing lung parenchyma, did not lead to important changes in oxygenation as compared to the other groups. Our findings agree with those of Andrews and colleagues, who demonstrated that rats ventilated during short-term with different positive end-expiratory pressures (PEEP) develop lung damage but maintain good oxygenation, suggesting that the assessment of oxygenation alone may not be a good marker of parenchymal damage [35]. Maruscak and colleagues also observed that oxygenation remained unchanged until 90 minutes of MV in rats ventilated with tidal volumes of 8 mL/kg or 30 mL/kg and PEEPs [36].

Mechanical ventilation has been associated with exacerbation of lung inflammation and impairment of pulmonary characteristics such as leukocyte infiltration and cytokine accumulation. Thus, the precise identification of biomarkers to evaluate ICU patients is wanted. Recently, we have shown that pressure-controlled ventilation in female Wistar rats promoted structural changes to the lung parenchyma and triggered redox imbalance and inflammation (settings: 1 h ventilation, VT of 8 mL/kg, ZEEP) [23].

Differential diagnosis of lung diseases is typically conducted by BALF analysis [37], and studies have already shown significant changes in BALF in ventilated patients with pulmonary lesions [38], as a release of proinflammatory cytokines, increased permeability, and influx of neutrophils, macrophages, and lymphocytes into the lung [23, 39, 40]. Although macrophages and lymphocytes are associated with MV and may promote the increase of proinflammatory cytokines, in our short-term MV experimental model, we demonstrated that in healthy animals submitted to mechanical ventilation, there was no significant variation in the macrophage and lymphocyte counts corroborating with our previous studies [21, 22]. On the other hand, in this work, healthy animals mechanically ventilated with a tidal volume of 12 mL/kg presented higher neutrophil counts in the BALF than CG animals. Neutrophils are the first defense cells recruited into the inflammatory focus [41], and, therefore, it is accepted that an impaired capillary-alveolar barrier may jeopardize vascular permeability and promote the activation of alveolar macrophages [42]. As a result, local inflammatory mediators are released and influx of neutrophils into the lung will ensue [10, 42]. Our results agree with those of Cagle and colleagues, who ventilated rats for 2 hours and disclosed a larger number of neutrophils in the BALF of those ventilated with a tidal volume of 15 mL/kg than the group ventilated with 8 mL/kg [43].

The cyclical stretch generated by artificial ventilation can induce damage and, subsequently, the influx of inflammatory cells and is associated with proinflammatory markers [9]. In this context, we observed an increase in IL-1, IL-6, and TNF-*α* levels in lung homogenates in animals that underwent MV with tidal volume of 12 mL/kg. Our data agree with those of Tremblay and colleagues, who report an increase in TNF-*α*, IL-1, and IL-6 levels in rats ventilated for 2 hours with large tidal volumes (40 mL/kg) without using PEEP [44]. In general, these cytokines are produced by several types of cells, such as alveolar macrophages [45], initiating physiological responses under different stimuli, which may result in excessive activation of the inflammatory cascade and in the overproduction of proinflammatory mediators [46]. Indeed, increased TNF-*α*, IL-1*β*, and IL-6 levels were detected in the BALF of patients with acute respiratory distress syndrome [46]. We demonstrated that MV with 12 mL/kg increased the neutrophil number in the airways and augmented the production and release of TNF-*α*, IL-1, and IL-6, stressing the strict control of ventilatory parameters under MV. These changes were not found in rats ventilated with smaller tidal volumes. Clinicians need to be aware of the potential risks of high tidal volume, such as inflammatory disorders conducting lung injuries even in a short time. Once in human patients it is difficult to measure lung parenchymal damage after one hour of MV, our findings could contribute to improve some ventilation parameters in physiopathological conditions such as infections in lungs where the inflammatory pattern observed is higher than healthy condition [47] and also avoid pulmonary injuries additional when high tidal volume is used in the disease status. Recently, a prospective observational study in COVID-19 patients admitted to ICU ventilated with a lung-protective strategy with a diagnosis of pneumonia secondary to SARS-CoV-2 infection was demonstrated that the IL-6, IL-1*β*, and TNF-*α* levels of serum were significantly higher in both COVID-19 and ICU control patients compared with healthy control values (non-COVID-19 critically ill), being that was observed a specific association between elevated IL-6 and severe COVID-19-associated lung injury [48]. In this context, assessment of the efficacy and safety of mechanical ventilation settings and treatments is the basis of the first phase of the management of patients; our findings have shown the impact of high tidal volume in controlled mode ventilation over redox imbalance and lung inflammation in healthy adult rats and could be relevant extremely to avoid complications possible in healthy or to worsen the clinic status in injured lungs, special attention to vent settings should be taken in the infection conditions.

On the other hand, the concentration of CCL5 diminished in animals ventilated with a tidal volume of 12 mL/kg in relation to the control group only. Since chemokines play an important role in cell recruitment, this initial response might be a homeostatic mechanism to avoid the severity of the disease. Several studies report that inhibition of CCL5 function reduces neutrophil activation, and high CCL5 expression correlates with neutrophil activation in lung disease [49, 50]. Huang and colleagues report that CCL5 serum levels fall in the acute phase of severe acute respiratory syndrome, suggesting the existence of downregulation mechanisms to minimize the inflammatory response [51].

Mechanical ventilation is associated with oxidative damage too. In this line, the prolonged use of MV may trigger diaphragmatic dysfunction, owing to a great extent to oxidative stress [52, 53]. Andrade and colleagues did not observe an increase in lipid peroxidation and protein oxidation in the lung parenchyma of healthy rats ventilated with a tidal volume of 7 mL/kg [22]. Moreover, Sun and colleagues reported an association between large tidal volumes (30 and 42 mL/kg, 2 h MV) and severe oxidative stress and antioxidant responses in Wistar rats [8]. In this study, we found higher degrees of lipid peroxidation and protein oxidation in animals ventilated with a tidal volume of 12 mL/kg than in spontaneously breathing and MVG4 rats, as well as an increase in MPO in the pulmonary homogenate. MPO is an oxidizing enzyme produced and stored by neutrophils and macrophages and released into the extracellular fluid in the scenario of the inflammatory process [54]; it is considered a hallmark of neutrophil activation and modulates lung epithelial responses to proinflammatory agents [55].

Twelve mL/kg ventilation produced an increase in SOD and CAT activities in pulmonary homogenates. SOD is an important antioxidant enzyme that protects cells against damage caused by the superoxide anion, since it catalyzes the dismutation reaction of superoxide radicals into molecular oxygen and hydrogen peroxide, while catalase is one of the enzymes responsible for reducing the hydrogen peroxide into water and oxygen [14]. Inflammatory cell infiltration, mainly neutrophils, increases the levels of reactive oxygen species (ROS) and, as a result, rises the activity of antioxidant enzymes in an attempt to minimize the damage caused by ROS in the lung parenchyma in the experimental model of VILI [56]. Wu and colleagues report that intravenous administration of SOD in rats displaying lung damage caused by ventilation with high tidal volume (18 mL/kg) reduces lung injury and oxidative stress [57]. In this context, Birben and colleagues describe an increase in antioxidant enzymatic activity in response to oxidative aggressions [14], which supports the evidence of regulation of redox status, protecting against prooxidant stimuli by means of the production of antioxidant enzymes such as superoxide dismutase and catalase. However, the increase in the activity of the measured antioxidant enzymes observed in our study did not prevent the oxidative damage observed in the ventilated group with 12 mL/kg.

In addition, to evaluate inflammatory biomarkers and oxidative stress, we assessed pulmonary morphology aiming at determining possible lung lesions that could directly influence gas exchange [58]. Stereologically, we found lung parenchymal damage after one hour of MV with 12 mL/kg, consisting of higher volume densities of the alveolar air space and smaller volume densities of alveolar septa than in the remaining groups. In this context, Izquierdo-Garcia and colleagues demonstrated injury to the endothelium and components of the extracellular matrix in ventilated rats with a high VT of 25 mL/kg [59], and Moraes and colleagues [60] observed damaged epithelium and endothelial cells due to MV with a VT of 22 mL/kg. MVG4 and MVG8 rats showed no differences in inflammatory and oxidative profile, as well as changes in pulmonary histoarchitecture compared to CG, which may suggest that the onset of ventilator-induced injury is correlated with the recruitment of inflammatory cells and their oxidative contents. Our study suggests that the stereological changes induced by high tidal volume in the pulmonary parenchyma were associated with oxidant agents damaging the lung. Our study for the first time measured pulmonary redox imbalance and determined its association with pulmonary inflammatory markers in a lung injury model induced by short-term mechanical ventilation in healthy rats.

In conclusion, this study showed that MV with normoxia (21% O_2_), ZEEP, and tidal volume of 12 mL/kg for one hour induced oxidative injury in healthy lungs, accompanied by neutrophil influx and inflammatory cytokines in the airways.

## Figures and Tables

**Table 1:** Blood gas analyses and lung function in Wistar rats.

Group	CG	MVG4	MVG8	MVG12
pH	7.39 ± 0.03	7.24 ± 0.04^a^	7.44 ± 0.03^b^	7.50 ± 0.04^a,b,c^
PO_2_ (mmHg)	77.71 ± 20.03	50.98 ± 6.93^a^	70.23 ± 9.40^b^	79.40 ± 14.60^b^
PCO_2_ (mmHg)	36.66 ± 5.93	64.16 ± 4.73^a^	29.60 ± 4.41^b^	16.83 ± 3.05^a,b,c^
HCO_3_^−^ (mEq/L)	22.77 ± 2.80	28.08 ± 2.42^a^	20.43 ± 1.48^b^	13.73 ± 1.83^a,b,c^
SO_2_ (%)	91.27 ± 6.26	76.80 ± 8.14	94.31 ± 2.61^b^	96.83 ± 1.42^b^
PO_2_/FiO_2_ (mmHg)	371.80 ± 95.85	243.70 ± 33.17^a^	336.0 ± 44.99^b^	379.90 ± 69.88^b^
RR (breaths/min)	88.00 ± 17.01	70.50 ± 1.69	70.50 ± 1.41	70.38 ± 1.30
VT (mL)	2.28 ± 0.65	1.37 ± 0.11^a^	2.76 ± 0.18^b^	4.15 ± 0.30^a,b,c^
V'min (mL/min)	182.90 ± 63.04	96.77 ± 5.74^a^	194.80 ± 8.87^b^	292.10 ± 15.95^a,b,c^

CG: control group; MVG4: mechanical ventilation group 4 mL/kg; MVG8: mechanical ventilation group 8 mL/kg; MVG12: mechanical ventilation group 12 mL/kg; PO_2_: partial pressure of oxygen; PCO_2_: partial pressure of carbon dioxide; HCO_3_^−^: bicarbonate; SO_2_: oxygen saturation; FiO_2_: inspired fraction of oxygen; RR: respiratory rate; VT: tidal volume; V'min: minute ventilation. ^a^Represents a significant difference in relation to CG. ^b^Represents a significant difference in relation to MVG4. ^c^Represents a significant difference in relation to MVG8. Data are expressed as mean ± standard deviation. Analysis of Variance One-Way ANOVA followed by Tukey's posttest (*p* < 0.05). *N* = 8 animals per group.

**Table 2:** Inflammatory markers in lung parenchyma.

Group	CG	MVG4	MVG8	MVG12
IL-1 (pg/mL)	468.9 ± 130.9	614.2 ± 151.7	776.8 ± 61.54	943.0 ± 276.3^a,b^
IL-6 (pg/mL)	1553.0 ± 135.7	1505.0 ± 130.1	1739.0 ± 54.7	1809.0 ± 120.2^a,b^
TNF-*α* (pg/mL)	1293.0 ± 187.1	1263.0 ± 85.7	1420.0 ± 119.2	1572.0 ± 113.4^a,b^
CCL5 (pg/mL)	526.9 ± 133.4	336.4 ± 155.4	328.3 ± 186.4	241.2 ± 66.86^a^

CG: control group; MVG4: mechanical ventilation group 4 mL/kg; MVG8: mechanical ventilation group 8 mL/kg; MVG12: mechanical ventilation group 12 mL/kg; IL-1: interleukin 1; IL-6: interleukin 6; TNF: tumor necrosis factor; CCL5: CC chemokine ligand-5 (CCL5/RANTES). ^a^Represents a significant difference in relation to CG. ^b^Represents a significant difference in relation to MVG4. Data are expressed as mean ± standard deviation. Analysis of Variance One-Way ANOVA followed by Tukey's posttest (*p* < 0.05). *n* = 8 animals per group.

**Table 3:** Biomarkers of oxidative stress in lung parenchyma of animals.

Group	CG	MVG4	MVG8	MVG12
TBARS (nmol/mg protein)	0.80 ± 0.20	0.71 ± 0.40	0.71 ± 0.11	1.74 ± 0.57^a,b,c^
PTN CARB (nmol/mg protein)	6.41 ± 1.73	6.88 ± 1.86	7.14 ± 1.57	17.69 ± 3.13^a,b^
SOD (U/mg protein)	18.59 ± 2.47	18.01 ± 7.07	25.32 ± 9.23	33.96 ± 15.57^a,b^
CAT (U/mg protein)	3.09 ± 0.96	3.48 ± 1.10	3.87 ± 0.49	5.39 ± 1.71^a^
MPO (Um/mg protein)	0.35 ± 0.11	0.60 ± 0.20	0.50 ± 0.12	1.34 ± 0.56^a^

CG: control group; MVG4: mechanical ventilation group 4 mL/kg; MVG8: mechanical ventilation group 8 mL/kg; MVG12: mechanical ventilation group 12 mL/kg; TBARS: thiobarbituric acid reactive substances; PTN CARB: protein carbonyl; SOD: superoxide dismutase; CAT: catalase; MPO: myeloperoxidase. ^a^Represents a significant difference in relation to CG. ^b^Represents a significant difference in relation to MVG4. ^c^Represents a significant difference in relation to MVG8. Data are expressed as mean ± standard deviation. Analysis of Variance One-Way ANOVA followed by Tukey's posttest (*p* < 0.05). *n* = 8 animals per group.

## Data Availability

The data obtained in this study are available from the corresponding author upon request.

## Abbreviations

BALF: Bronchoalveolar lavage fluid
FiO_2_: Fraction of inspired oxygen
MV: Mechanical ventilation
PaCO_2_: Arterial partial pressure of carbon dioxide
PaO_2_: Arterial partial pressure of arterial oxygen
VILI: Ventilator-induced lung injury
V'min: Minute volume/volume
VT: Tidal volume.
